# First person – Christina Walker

**DOI:** 10.1242/bio.061689

**Published:** 2024-09-05

## Abstract

First Person is a series of interviews with the first authors of a selection of papers published in Biology Open, helping researchers promote themselves alongside their papers. Christina Walker is first author on ‘ Mutational cooperativity of RUNX1::RUNX1T1 isoform 9a and oncogenic NRAS in zebrafish myeloid leukaemia’, published in BiO. Christina is a research assistant in the lab of Dr Andrew Wood at the University of Auckland, New Zealand, investigating the interplay between genetic alterations to drive cancer and how precision medicine can help inform progression, prognosis and treatment.


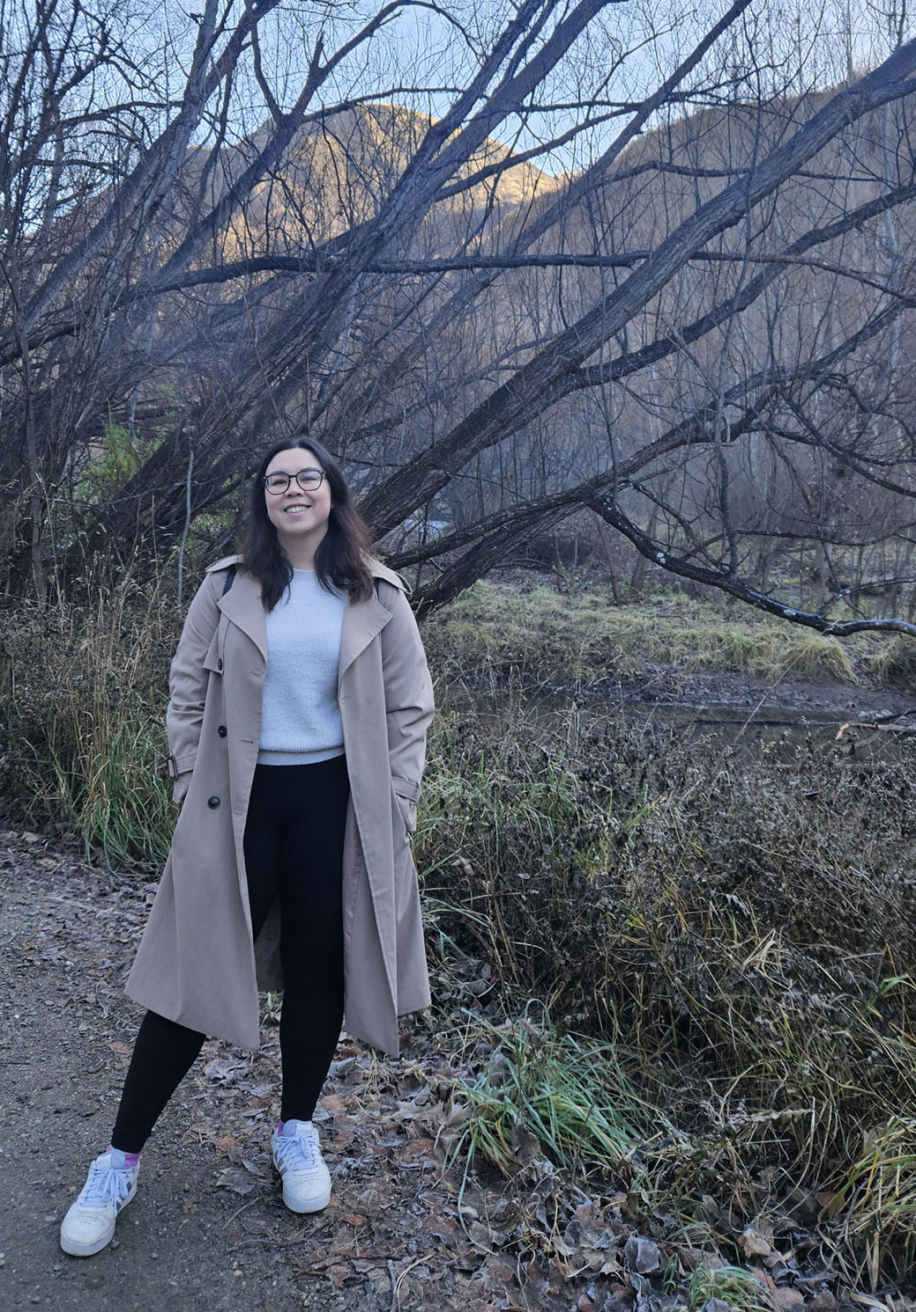


**Christina Walker**

**Describe your scientific journey and your current research focus**

My undergraduate years were focused on genetics and biochemistry to better understand the basis of life and mechanisms of disease. My postgraduate journey is where I've found my love for research using *in vitro* systems to look at protein function and protein production for crystallisation. Since joining the Wood Lab I've been able to combine a multi-faceted approach with *in vitro* systems to look at protein expression, protein-protein interactions, drug treatments; learning and helping to develop our AML zebrafish model, and I've been able to relate this to patient genetics using variant curation to better understand the mutational landscape of paediatric cancers.

…and like many I have had family members diagnosed with cancer and watched their battle

**Who or what inspired you to become a scientist?**

Looking down a light microscope as a child I fell in love with biology, and like many I have had family members diagnosed with cancer and watched their battle. It felt natural to pursue a career in molecular biology with a focus on cancer to gain better understanding of cancer mechanisms in the hope we could uncover information that could change the lives of cancer patients.

**How would you explain the main finding of your paper?**

As DNA replicates, errors (genetic variants) can be introduced that can give cells “superpowers” to become cancerous – by making them better at dividing, spreading, and evading cell death. Some of these work as a team to make cells malignant. Remarkably, we showed that only TWO mutations were enough and we showed that they also caused AML in fish!

**What are the potential implications of this finding for your field of research?**

Our model helps to explain the phenomenon seen in newborns without an AML diagnosis but have t(8;21), and then at diagnosis where many patients also have a number of other known driver mutations. We showed that 9a alone (t(8;21)) is not enough to cause cancer but requires the help of NRAS^G12D^ in order to drive AML, and that just two mutations were enough for disease. So now we can work towards unravelling their game plan to better understand disease progression and look at identifying other key players for cooperation to drive disease and possibly even identify targets.

**Figure:**
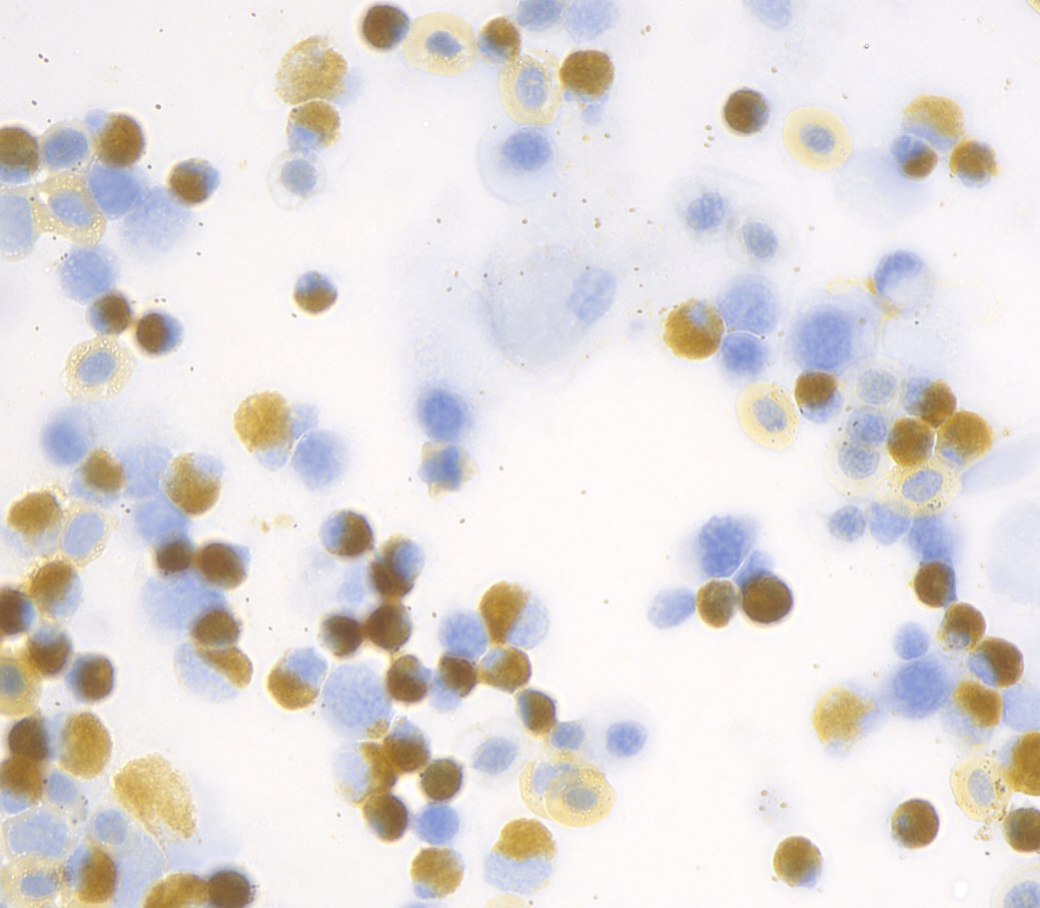
**Myeloperoxidase activity visualised in zebrafish haematopoietic cells using 3, 3′ diaminobenzidine (3, 3′ DAB).** This is converted by the enzyme to a golden-brown substrate. Marking myeloid lineage cells and uniform staining of the extracellular compartment of erythrocytes.

**Which part of this research project was the most rewarding?**

Having had limited experience with animal models prior to this project, the most rewarding part was moving from 2D cell culture to showing how the two genetic alterations interacted to drive a spectrum of AML phenotypes in zebrafish – mirroring what can be seen in patients.

**What do you enjoy most about being an early-career researcher?**

I love the ability to work independently but with the knowledge that there are people more knowledgeable and wiser than I am, that I can ask for help and advice.

**What piece of advice would you give to the next generation of researchers?**

Understanding that a failed experiment does not mean you failed but that it's another clue in the puzzle, and that passion for your research topic is a great driver.

**What's next for you?**

Up next is using the skills I learnt working with zebrafish to better understand other oncofusions and how they drive paediatric cancers. In collaboration with other researchers and clinicians I want to apply variant curation, precision medicine and drug screens to develop model systems with the goal of helping paediatric patients (identifying prognostic variants, targeted therapies and biomarkers).
